# Editorial: Adverse and Toxic Effects of Childhood Cancer Treatments

**DOI:** 10.3389/fonc.2021.795664

**Published:** 2021-11-03

**Authors:** Antonio Ruggiero, Roderick Skinner, Wael Zekri Khaled Zekri

**Affiliations:** ^1^ Pediatric Oncology Unit, Fondazione Policlinico Universitario Agostino Gemelli Istituto di Ricovero e Cura a Carattere Scientifico (IRCCS), Università Cattolica del Sacro Cuore, Rome, Italy; ^2^ Department of Paediatric and Adolescent Haematology/Oncology, Great North Children’s Hospital, Newcastle, United Kingdom; ^3^ Newcastle University Centre for Cancer, University of Newcastle, Newcastle, United Kingdom; ^4^ National Cancer Institute, Cairo University and Children Cancer Hospital, Cairo, Egypt

**Keywords:** children, adverse effects, cancer, treatment, fertility

The development of effective treatments for cancer has been a landmark achievement of medical research in the modern era. Now, many children with cancer can be cured with dramatic increases in the survival rates of most types of childhood cancers over the last 30 years (1). Most antineoplastic drugs can produce acute toxicities (i.e., nausea, vomiting, mucositis, alopecia,…) which are generally reversible once the chemotherapy is completed (2–4). Some anticancer drugs are associated with delayed toxic effects which can became evident even many years from the chemotherapy (5, 6). Therefore, as the number of survivors from childhood cancers is increasing, late effects are now becoming major concerns highlighting the importance of long-term follow-up (7, 8).

This Research Topic includes 12 manuscripts focusing on the pathophysiology, diagnosis, treatment, and outcomes of acute and long-term side effects in children treated for cancer.

In their retrospective study, Lu et al. aimed to investigate the relationships between the methylenetetrahydrofolate reductase (MTHFR) C677T/A1298C and high-dose methotrexate (HD-MTX)-related toxicities in a homogenous group of children with non-Hodgkin lymphoma (NHL). Patients harboring mutant C677T genotype were more vulnerable to oral mucositis, leucopenia, and thrombocytopenia while those with mutant A1298C genotype were more likely to develop anemia and leucopenia but less susceptible to vomiting. These results can be of great interest for clinicians as predictors of MTX toxicity can help to determine the appropriate individual dosage minimizing adverse effects.

Attinà et al. reported their experience of the management of oral mucositis as well as documenting its risk factors. Mucositis was a common complication of treatment for childhood malignancies with 50% of patients suffering from at least one episode. Its occurrence was related to the presence of neutropenia, number of chemotherapy courses, and type of tumor. The WHO oral mucositis scale appeared to be a valuable tool for assessing the severity of mucositis and the relative effectiveness of pain relief with opioids.

The study by Blom T et al. provides a focus on the treatment-related toxicities during immunotherapy with dinutuximab, IL-2, GM-CSF, and isotretinoin in 26 high-risk neuroblastoma patients receiving treatment according to the DCOG NBL2009 protocol. The most common grade ≥3 toxicities were pain, central venous catheter-related infections, and fever. In total, 310 grade ≥3 toxicities were recorded in 124 courses and a higher number of toxicity episodes was observed in children receiving IL-2.

With a broad perspective on its potential for cancer treatment, the review by Cerchione et al. outlines the role of Dasatinib in the management of childhood Philadelphia chromosome-positive acute lymphoblastic leukemia (Ph+ALL). The use of the second generation tyrosine kinase inhibitors (TKIs), such as Dasatinib, has significantly improved the outcome for pediatric patients with Ph+ALL when used in combination with the standard chemotherapy protocols. The role of Dasatinib in combinations with other molecules for new risk-adapted/MRD-driven clinical trials in relapsed Ph+ALL.or in a post hemopoietic stem cell transplantation setting has to be investigated in future studies.

Several articles within this Research Topic explore recent developments in fertility preservation. Brancati et al. underline the variations in access to available preventive measures. Pharmacological treatment with GnRHa has become an alternative non-invasive and well-tolerated approach, especially in those who cannot access cryopreservation options due to clinical and/or logistic issues. Nevertheless, at present, only very limited evidence from randomized clinical trials on the use of GnRHa in adolescents with cancer is available. In their translational research model, Hao et al. explore the feasibility of follicle isolation and culture from prepubertal mice ovaries recently treated with cyclophosphamide as an alternative fertility preservative method. In addition, the reproductive potential of these follicles regarding the final achievement of mature competent oocytes has been also studied. The follicles obtained were able to grow, and had spindle and chromosome configurations that were normal in the mature oocytes. Wikander et al. report their prospective study involving prepubertal and adolescent girls undergoing HSCT and the outcomes of fertility preservation treatments performed before or after HSCT. Although, the study has some limitations due to the small size, authors were able to demonstrate that fertility preservation can be achieved before and also after HSCT so enhancing the chances of future pregnancies.

Camet et al. assessed the effect of dosing, infusion times, and schedules of cisplatin administration on hearing loss in children receiving cisplatin. Their findings show that the amount of cisplatin infused per dose was strongly associated with an increased risk of hearing loss as well as cumulative cisplatin dose and young age at treatment.

Evidence is accumulating that for childhood cancer survivors, late effects including cardiotoxicity, chronic health conditions, neurocognitive and behavioral functioning can have a negative impact on their quality of life. Blom JM et al. suggest the adoption of digital phenotyping and dynamic monitoring for adolescents treated for cancer. Their integrated multi-dimensional model, as part of a digital toolbox, can represent a new approach for the development of age-appropriate resources that could help them in managing their disease and the treatment side effects. The work of Sofia et al. aimed at detecting late subclinical cardiac dysfunction in children treated for cancer with anthracyclines. The early recognition of such sub clinical cardiac dysfunction may be fundamental for facilitating prompt appropriate medical management. They did not identify statistically significant correlations between echocardiographic parameters (2D, strain and 3D assessment) and age at cancer diagnosis or duration of follow-up. Significantly reduced 3D left ventricle ejection fraction was reported in children treated with anthracyclines despite no significant differences in 2D ejection fraction and longitudinal strain values.

Ewig et al. conducted a retrospective study on chronic polypharmacy prescription among childhood cancer survivors. They confirm the potentially high incidence of late, and often permanent, health complications arising from intensive treatment with combination chemotherapy and ionizing radiation especially for children with central nervous system tumors or survivors who undergone hematopoietic stem cell transplantation. For physicians who are dedicated to taking care of these patients, future work requires appropriate consideration for this vulnerable population.

In their prospective study on Chinese survivors of childhood acute lymphoblastic leukemia (ALL), Peng et al. detected that most young survivors had normal cognitive and behavioral function during the early phase of survivorship although subjects with chronic health conditions or negative socio-environmental were at higher risk of executive cognitive dysfunction.

Although the studies presented in this Research Topic cannot comprehensively cover the whole breadth of this topic, the result of each individual study opens new research and clinical questions helpful to build a new health system model aimed at reducing the risk of major adverse and toxic effects related to cancer treatment.

## Author Contributions

AR conceived the draft. RS and WK contributed to the manuscript text and editing. RS supervised the manuscript writing process. All authors provided critical feedback and helped shape the direction of the manuscript.

## Conflict of Interest

The authors declare that the research was conducted in the absence of any commercial or financial relationships that could be construed as a potential conflict of interest.

## Publisher’s Note

All claims expressed in this article are solely those of the authors and do not necessarily represent those of their affiliated organizations, or those of the publisher, the editors and the reviewers. Any product that may be evaluated in this article, or claim that may be made by its manufacturer, is not guaranteed or endorsed by the publisher.
